# Caregiver decision-making concerning involuntary treatment in dementia care at home

**DOI:** 10.1177/09697330211041742

**Published:** 2021-12-06

**Authors:** Vincent RA Moermans, Angela MHJ Mengelers, Michel HC Bleijlevens, Hilde Verbeek, Bernadette Dierckx de Casterle, Koen Milisen, Elizabeth Capezuti, Jan PH Hamers

**Affiliations:** Maastricht University, The Netherlands; White Yellow Cross Limburg, Belgium; Living Lab in Ageing and Long-Term Care, the Netherlands; Maastricht University, The Netherlands; Living Lab in Ageing and Long-Term Care, The Netherlands; KU Leuven, Belgium; University Hospitals Leuven, Belgium; Hunter College and City University of New York, USA; Maastricht University, The Netherlands; Living Lab in Ageing and Long-Term Care, The Netherlands

**Keywords:** Autonomy, ethics and dementia care, family caregivers, home care, involuntary treatment, safety

## Abstract

**Background:**

Dementia care at home often involves decisions in which the caregiver must weigh safety concerns with respect for autonomy. These dilemmas can lead to situations where caregivers provide care against the will of persons living with dementia, referred to as involuntary treatment. To prevent this, insight is needed into how family caregivers of persons living with dementia deal with care situations that can lead to involuntary treatment.

**Objective:**

To identify and describe family caregivers’ experiences regarding care decisions for situations that can lead to involuntary treatment use in persons living with dementia at home.

**Research design:**

A qualitative descriptive interview design. Data were analysed using the Qualitative Analysis Guide of Leuven.

**Participants and research context:**

A total of 10 family caregivers providing care for 13 persons living with dementia participated in in-depth semi-structured interviews. Participants were recruited by registered nurses via purposive sampling.

**Ethical consideration:**

The study protocol was approved by the Ethics Committee of the University Hospitals Leuven and the Medical Ethical Test Committee Zuyderland.

**Findings:**

Family caregivers experience the decision-making process concerning care dilemmas that can lead to involuntary treatment as complicated, stressful and exhausting. Although they consider safety and autonomy as important values, they struggle with finding the right balance between them. Due to the progressive and unpredictable nature of dementia, they are constantly seeking solutions while they adapt to new situations. Family caregivers feel responsible and experience social pressure for the safety of persons living with dementia. They may be blamed if something adverse happens to the persons living with dementia, which increases an already stressful situation. Their experience is influenced by characteristics of the care triad (persons living with dementia, professional and family caregivers) such as practical and emotional support, knowledge, and previous experiences.

**Discussion and conclusion:**

To prevent involuntary treatment, professionals need to proactively inform family caregivers, and they need to support each other in dealing with complex care situations.

## Introduction

Worldwide, dementia has a profound health impact on those who have it and on their caregivers as well. The number of persons living with dementia (PLWD) worldwide will triple from 35.6 million to 115.4 million by 2050.^1^ The majority of PLWD age in place, and wish to live there as long as possible, making family caregiving a major portion of dementia care provision.^1,2^ However, caring for PLWD can be stressful and difficult to manage for family caregivers. Dementia involves progressive loss of mental and physical abilities^2^ and decision-making capacity,^3^ and behavioural and psychological symptoms which can lead to caregiver burden.^4^

When family caregivers perceive that the cognitive skills of PLWD decline to a point, where they find that the PLWD are no longer able to make decisions about everyday life themselves, they gradually take a dominant role in these decisions.^5,6^ Making proxy decisions is complex and can involve ethical dilemmas for family caregivers. The needs and wishes of family caregivers and PLWD can differ, especially since the PLWD may not appreciate their vulnerabilities.^7,8^ Family caregivers want to ensure a safe environment because they regard their family member as vulnerable to potential dangers, such as (injurious) falls, getting lost, or health problems due to insufficient body hygiene or incorrect medication intake.^8–11^ According to Kitwood,^12^ PLWD are in need of comfort, attachment, inclusion, occupation and identity, which form the basis of person-centred care (PCC). The PCC approach enables caregivers to understand, and provide support for, the unmet needs and wishes of the person receiving care.^13^ However, family caregivers may find it essential to take safety measures^7–9^ that may lead to situations to which PLWD resist.^14^ Family caregivers do not always have sufficient skills, knowledge or support to respond to care for resistance^15–17^ in a manner that is aligned with a PCC approach.^13^

Several terms are used in the literature to describe care to which PLWD resist or do not provide consent for, such as coercive care,^17^ restiveness to care^18^ and involuntary treatment.^19,20^ This study uses the term ‘involuntary treatment’, defined as care provided without the consent of the person receiving it and/or to which this persons resists. Involuntary treatment includes the use of physical restraints, psychotropic medication and non-consensual care.^19^ Recent research^21^ shows that one out of two PLWD living at home receive involuntary treatment, mostly requested and used by family caregivers and nursing staff. Also, family caregivers are more accepting of involuntary treatment use than professional caregivers.^22^ Commonly reported reasons to apply involuntary treatment are safety related such as protection of the PLWD and/or his environment.^10^ However, involuntary treatment can be considered as inappropriate because it can have negative influence on the physical and psychological well-being of the PLWD,^23^ and often alternative interventions exist that are less restrictive, safer, more effective and in line with PCC.^13^ In addition, involuntary treatment is associated with higher care burden by family caregivers.^21^

In order to prevent and/or reduce involuntary treatment use in PLWD living at home, more insight is needed into how family caregivers deal with and make decisions in care situations that can lead to involuntary treatment. These insights can be used to develop an individualised, person-centred approach to support family caregivers in dealing with these dilemmas. This study aims to identify and describe family caregivers’ experiences regarding care decisions concerning situations that can lead to involuntary treatment use in PLWD at home.

## Methods

### Design

To perform a straight description of the experiences from family caregivers of PLWD and to stay close to the findings, we used a qualitative descriptive research design.^24^

### Setting and sampling

Data were collected between November 2019 and February 2020 among family caregivers of PLWD receiving professional dementia care at home. ‘Family caregiver’ was broadly defined as a non-paid caregiver who has a significant emotional relationship with the PLWD. This could be a family member or friend, who offers emotional-expressive, instrumental and tangible support and assistance to PLWD.^25^ Participants were selected through maximum variation sampling.^24,26^ The recruiters purposefully approached those family caregivers who had rich experiences with care situations regarding safety versus autonomy, which might have or actually led to involuntary treatment, and the recruiter informed them using an information letter. The main inclusion criteria were: (1) being a family caregiver for a PLWD living at home, (2) Dutch speaking and (3) having experience with dealing with care dilemmas that could lead to involuntary treatment. If the family caregivers were interested in the study, they were contacted by the researchers to further inform them and plan an appointment to conduct the interview. In addition, based on the insights during the data analysis, we purposefully contacted family caregivers based on their demographic characteristics such as, age, gender, relation to PLWD, living together with PLWD and the use of involuntary treatment. The research team has experience with care for older people, home care nursing, dementia care, falls prevention, involuntary treatment, (physical) restraint use, qualitative research and the Qualitative Analysis Guide of Leuven (QUAGOL).^27^

### Data collection

Interviews were conducted by the principal researchers (V.M. and A.M.) at the participants’ home or researchers’ office. All interviews were conducted in Dutch, audio-recorded with participants’ permission and transcribed by the principal researchers. It was anticipated that interviews would last approximately 60 min. The interviews were performed using an informal interview technique including an open and broad conversation focusing on participant experiences. Spontaneous follow-up questions were asked during the interview. The interview guide is presented in Table 1. After each interview, the researcher took field notes documenting the details of the observations and the process of interactions. After eight interviews (describing 11 cases of PLWD) were conducted, results were discussed with the research team. To further enrich data and reach saturation, two more interviews were conducted. The results of the two last cases confirmed the themes without any new or additional themes or information. Since redundancy was achieved, the research group decided that data saturation was met. The final sample consisted of 10 family caregivers (8 female) who cared for 13 PLWD at home (7 female). Three participants had experience with providing family care for two PLWD; thus, data were collected from 13 care situations with PLWD. The average age of the family caregivers was 58 years (range: 44–70 years) and of the PLWD, 73 years (range: 59–90 years). In 7 of the 13 cases, the family caregiver was the partner, and in 6 cases, it was the daughter/son. Tables 2 and 3 provide an overview of the family caregivers’ and PLWD’s characteristics. Table 4 shows family caregivers’ experiences with the use of involuntary treatment or alternatives. In 10 of the 13 cases, at least one type of involuntary treatment was applied.

**Table 1.** Interview guide.

What is your experience and knowledge in caring for PLWD? What do you find important in the care for your loved one? Tell me about the first time that you were confronted with the fact that your opinion about safety differs from the person you care for? How did you deal with it? How did this effect you? How did that effect your environment? Who was involved in care of your loved one? What support did you receive from others (family, professional caregivers) in dealing with these situations? How did you experience this support? Follow-up question: Can you tell me more about it? What happened next? What were you thinking then? How did that effect you? How did you feel? What do you mean by that?

PLWD: persons living with dementia.

**Table 2.** Family caregivers’ personal characteristics (N = 13).

Item	Response	Amount
Gender	Male	4
	Female	9
Age	30–39	1
	40–49	1
	50–59	5
	60–69	5
	70–79	1
Education	Associate’s degree	5
	Bachelor’s degree	7
	Master’s degree	1
Experience as family caregiver (years)	0–2	4
	3–4	3
	5–6	3
	7–8	2
	9–10	1
Relationship to PLWD	Partner	7
	Daughter/son	6

Because three family caregivers provided care for two PLWD, the total is 13. PLWD: persons living with dementia.

**Table 3.** Personal characteristics of PLWD (N = 13).

Item	Response	Amount
Gender	Male	6
	Female	7
Age	50–59	1
	60–69	4
	70–79	5
	80–89	2
	90–99	1
Living situation	Alone	3
	With the caregiver	10
Resistance	No resistance mentioned	3
	One or more instances	10
Perceived safety risks	Getting lost	5
	Falling	4
	Physical aggressive	3
	PLWD could not be left alone	3
	Other (e.g. inappropriate medication intake, injury through sharp objects, traffic accidents, cooking accidents)	7
Mentioned safety incidents	Getting lost	6
	Falling	3
	Physically aggressive	2

Because three family caregivers provided care for two PLWD, the total is 13. PLWD: persons living with dementia.

**Table 4.** Involuntary treatment measures and alternatives.

Item	Response	Amount
Involuntary treatment: Physical restraints	Waist belt in a (wheel) chair	1
Psychotropic medication	Antidepressants	1
	Anti-psychotics	2
Non-consensual care	Forced hygiene	9
	Locking older adult in house	6
Alternatives		
	Enhance supervision	5
	Home adaptation	6
	Distracting PLWD	5
	Doing activities together	7
	Wait and see attitude (not intervening immediately)	8
	Engage in dialogue with PLWD	7
	Frequent calls/visits with PLWD	4

PLWD: persons living with dementia.

### Data analysis

Data analysis was based on QUAGOL,^27^ an iterative guidance tool for qualitative data analysis consisting of two parts: (1) the preparation for the coding process by paper-and-pencil work and (2) the actual coding process using qualitative software. During the first part, three researchers (V.M., A.M., M.B.) and a research assistant applied a case-oriented approach and identified the essential and common themes throughout the data. First, the researchers (re)read the transcripts individually and thoroughly, then developed a list of preliminary themes. Similarities, differences and connections among different themes within and across individual conceptual schemes were discussed by the four researchers. Using the method of constant comparison, they eventually found potentially relevant themes that can be used as codes. On the basis of this code list, all data were coded with qualitative software (MaxQData 2020®). All data were coded by linking each fragment of text to one of the themes from the preliminary code list. This resulted in a list of isolated themes and their meaning and characteristics. This list was discussed by the four researchers in the group in response to the research question. Then they distilled the storyline from the findings and themes. The final findings were discussed as a group and then submitted to the research team (V.M., A.M., M.B., H.V., J.P.H.) to reach consensus. They also discussed if data saturation was met.

### Ethical considerations

The Ethics Committee of the University Hospitals Leuven (reference G-2019 09 1735 on 10 July 2019) and the Medical Ethical Test Committee Zuyderland (reference METCZ20190118 on 12 September 2019) approved the study. All family caregivers received written and verbal information about the study. Before each interview, the participant filled in a consent form. Participation was on a voluntary basis and participants were free to withdraw at any time. Only the interviewers knew the participants’ identities. Data of participants were anonymised after transcription and treated confidentially.

### Rigour/trustworthiness

To ensure the trustworthiness of the study, several strategies were used^28^: (1) We maintained a detailed audit trail such as interview transcripts and field notes; (2) Thick description (i.e. relevant citations to illustrate the generated themes); (3) We performed member checking by summarising participants’ responses at the end of each interview; (4) The process of analysis was frequently reviewed within the research team to establish uniformity in themes and relationships and to explore the interviewers’ reflexivity; (5) Peer group discussion: the results were discussed with two district nurses specialised in guiding PLWD and their family caregivers; they recognised the themes from their own practice and acknowledged the findings of this study; (6) Triangulation such as constant comparison, case-oriented approach, open coding techniques; (7) Persistent observation; and (8) Prolonged engagement (i.e. researchers’ experience and duration of data collection).

## Results

The interviews revealed that family caregivers experience the decision-making process concerning care dilemmas that can lead to involuntary treatment use as complicated, stressful and exhausting. This was due to (1) the constant trade-off between safety versus autonomy, (2) constantly adapting and being prepared and (3) feeling responsible. How family caregivers experienced this decision-making process was influenced by characteristics of the care triad (PLWD, professional and family caregivers).

### Trade-off between safety versus autonomy

All family caregivers indicated that they found safety and autonomy important aspects in the care for PLWD. As long as the PLWD had no severe behavioural problems, safety risks or incidents, they supported the autonomy of the PLWD. Autonomy was described as involving PLWD in the decisions being made and supporting the PLWD to live a pleasant and meaningful life in their own home. As the mental and physical capabilities of PLWD deteriorate, more safety incidents often arise such as getting lost or injurious falls. As a result, family caregivers experienced that their and the PLWD’s opinions started to differ and they reported struggling with finding the right compromise between safety and autonomy. This was due to the increased safety risks experienced when respecting the wishes of the PLWD. At a certain point, often after an incident such as an injury or police intervention, safety outweighed the wishes of the PLWD. In some cases, this led to involuntary treatment (see Table 4) and/or the request for (more) professional help. Family caregivers indicated that this constant trade-off between safety and autonomy continuously affected their decisions and made it complicated and stressful to decide what was best. In care situations where involuntary treatment was applied, such as locking inside a house, the use of physical restraints or psychotropic medication, the family caregiver did not mention the negative impact of their actions such as feelings of imprisonment on the PLWD, and they justified them by saying that it was necessary to prevent falling and to reduce their own stress. On the contrary, in care situations where it was not applied, the family caregivers indicated that it was a deliberate choice not to intervene. For example, some accepted possible safety risks such as wandering and so they did not lock the PLWD in the house. They felt the safety risk did not outweigh the loss of dignity.

At some point, my stepmother started to deteriorate rapidly, she did not recognise my father anymore. She also started collecting scissors and all kinds of sharp objects in their bed and bedroom. At a certain point, we decided for my father’s safety that it couldn’t go on like this anymore. (50-year-old stepson of 77-year-old PLWD)

### Constantly adapting and being prepared

Family caregivers reported that due to the progressive and unpredictable nature of dementia, including behavioural changes and ongoing cognitive decline, they must constantly seek solutions that support a balance between safety and autonomy. However, these solutions, such as locking windows or doors, were limited and only applicable for a short period of time until new problems arose that could lead to involuntary treatment. Therefore, most of the family caregivers constantly needed to reflect on the situation and indicated that it was difficult to prepare for and adapt to unpredictable situations and found it hard to continuously look for ‘new solutions’. This combination of constantly seeking solutions and adapting to new situations makes dealing with these care situations challenging, exhausting and stressful. In contrast, a few family caregivers indicated that they did not anticipate what would happen and instead, lived in the moment.

The decline occurs in stages. One moment you think now I have found it, and the next moment there is something else again, and then you have to adapt to that. This is so exhausting that it breaks you. (67-year-old wife of 78-year-old PLWD)

Three weeks ago, I had the impression that I could get through to her, that she understood me and that she could be still alone at night. I went home with peace of mind, and that is not the case anymore. (51-year-old daughter of 80-year-old PLWD)

### Feeling responsible

Family caregivers mentioned that they felt obliged to take up the role as the primary caregiver for several reasons: (1) they could give back what their loved one had given them, (2) an earlier promise, (3) it was expected or (4) it was an expression of their love for the PLWD. They indicated that being the primary caregiver for the safety and well-being of their loved one was hard to bear. They were often worried about what could happen, especially if previous experiences in which they respected the wishes of PLWD (e.g. going for a walk or living alone) led to safety risks or incidents. They felt responsible ‘24/7’, meaning they were constantly on the alert for any problems. This resulted in both inadequate time to recover and persistent exhaustion. However, most of them also indicated they did not want to trouble, for instance, children and friends with their problems because they had their own life. For those who shared responsibility with others, decisions were made together, and the family caregiver felt supported, resulting in less burden and stress. But in some cases, family caregivers indicated that opinions regarding safety and autonomy differed between relatives, which they perceived as social pressure. Most family caregivers indicated that they were making plans and preparing themselves for the moment when would need to reject the wishes of the PLWD for safety reasons. By making these plans, they felt they could justify their decisions to tolerate safety risks, to themselves and others. Dealing with this social pressure to ‘do the right thing’, in combination with the feeling of possibly being blamed, was described as stressful and a great responsibility.

He [my partner living with dementia] does feel the need to go out alone. But because he has already fallen several times and people have called me about this, I’m afraid to let him go out alone. I’m afraid he’ll fall at some point and maybe break his neck. (70-year-old partner of 72-year-old PLWD)

I would like that he [my partner with dementia] live as long as possible in his own house, but I’m afraid that something could happen. I will feel guilty then, although I shouldn’t feel guilty because I offered him to come live with me. (57-year-old partner of 61-year-old PLWD)

### Characteristics of the care triad

Data analyses showed that family caregivers’ experiences with dealing with care dilemmas that can lead to involuntary treatment are influenced by several background characteristics of the care triad, including the PLWD, professional and family caregivers.

#### Characteristics of the PLWD

Family caregivers mentioned several patient-related characteristics that influenced their experience, including (1) changes in the PLWD’s behaviour (e.g. aggression, irritation, disorientation), (2) blurred boundaries between behaviour that arises from someone’s character versus the disease, (3) the frequency and severity of safety incidents and (4) the personality of the PLWD (i.e. the PLWD followed their advice and did not resist due to their docile nature).

#### Characteristics of the family caregivers

Family caregivers revealed that several characteristics influenced the way they dealt with care situations including (1) previous experience with providing care for PLWD, (2) their knowledge of alternatives (see Table 4), (3) their relationship to the PLWD, (4) their professional status (e.g. retired, employed) and (5) their coping strategies. Family caregivers who provided care for a parent with dementia in the past and had experience with involuntary treatment use indicated that they felt it was important to respect the autonomy and wishes of the PLWD. Family caregivers with knowledge of dementia, those that know how to manage behavioural symptoms and are aware of alternative measures experienced less difficulty dealing with care situations that could lead to involuntary treatment. Their relationship to the PLWD and living arrangement also influenced their experience. All family caregivers who did not live with the PLWD indicated that they could leave the situation and better cope with the stress than if they lived together. Family caregivers found it hard to balance the care for PLWD with their professional work and some took sick leave because they felt too stressed. Finally, family caregivers felt less stress if they regularly took rest breaks (e.g. use of a volunteer sitter or day care), or reduced the amount of time of in-person assistance such as checking on the PLWD by phone or by providing care either in the evening or morning.

#### Professional support

Family caregivers indicated that they needed more emotional support in the decision-making process from professional caregivers (listed in Table 5) because professionals (1) often underestimated the severity of caring for a PLWD, (2) lacked the time or knowledge to support them or (3) support was too late or too early. In addition, it took family caregivers much effort and time to find out what kind of support was available and they preferred more individualised information regarding their rights and possibilities to receive (professional) support. However, several family caregivers cited that they did not want or need professional support because (1) they wished to keep the care completely in their own hands, (2) providing care was still feasible, (3) they knew their loved one best and did not see how professionals could support them or (4) they did not want to bother others or ask for help. Instead, they sought solutions from the Internet and books and by talking to others in the same situation. In contrast, other family caregivers indicated that practical support from professionals was helpful and valuable. This support included (1) explaining the behaviour symptoms of PLWD and providing approaches to manage difficult situations, (2) providing care at home or day care so family caregivers had some respite from their care responsibilities and (3) medical management from the general practitioner (e.g. prescription of medication).

I think more guidance would have been an advantage and it would have helped. But on the other hand, I think you will also receive a lot of information that is not relevant in my case, because you are going to receive a lot of general information. I think in many cases it would be good to receive a little more guidance. (64-year-old wife of a 67-year-old PLWD)

**Table 5.** Professional and informal caregivers supporting primary caregiver.

Item	Response	Amount
Professional caregivers		
	General practitioner	7
	Dementia centre	8
	District nurse	8
	Psychologist	4
	Specialist physician	8
	Dementia case manager	2
	Day care for PLWD	2
	Domestic worker	3
Informal caregivers		
	Family or neighbours	10
	Volunteer sitters	2
	Others in same situation, support groups	9

PLWD: persons living with dementia.

#### Informal support

Informal support (listed in Table 5) included both practical assistance and emotional support. All family caregivers indicated they feel emotionally supported by talking with family and friends about their situation and sharing their experiences. The practical support included help with care-related tasks or going for a walk with the PLWD, which provided respite for the family caregivers. Most family caregivers attended caregiver support groups, which were recognised as very supportive because they could share experiences and advice in managing care dilemmas that could lead to involuntary treatment. The latter included the use of alternative measures such as a GPS-tracker, involvement of home care nursing or adaptations in the environment.

I am now part of a support group for family caregivers of PLWD and you learn a lot there. There are severe cases, very serious, but also light cases and you hear stories from everyone. When I leave these meetings, I go home with peace of mind and think to myself, oh but I am not that far yet, she is still an easy one. (67-year-old husband of a 68-year-old PLWD)

## Discussion

To our knowledge, this is the first study that focuses on the experiences of family caregivers regarding care decisions that could lead to the use of involuntary treatment. The current study links and confirms results from previous research regarding the decision-making process in dementia care at home,^3,6,9^ with the use of involuntary treatment. We provided new insights into how family caregivers experience this and how balancing safety with autonomy could evolve into the use of involuntary treatment. We found that the constant struggle with balancing safety with autonomy, constantly searching for solutions, adapting to new situations and being responsible for their safety was experienced as a complicated, stressful and exhausting process. Depending on the characteristics of the care triad, knowledge and experience regarding dementia and involuntary treatment use, the associated behavioural symptoms of PLWD and the received support, dealing with safety versus autonomy could all lead to the application of involuntary treatment.

We found that family caregivers recognised the need for autonomy of the PLWD and that they felt responsible to respect their wishes. However, at a certain point in the caregiving process, family caregivers experienced that their own needs and those of the PLWD’s regarding safety and autonomy started to differ and reported struggling with finding the right compromise between them, and conflicts thus started to arise. The ethical framework provided by Joan Tronto^29^ describes caregiving as a complicated, holistic process that does not always occur in a perfect way or end in ‘good care’, due to inherent conflicts between the needs of the caregivers and PLWD regarding attentiveness, responsibility, competence and responsiveness. We found these conflicts in our results as well. Several family caregivers mention that out of filial obligation^30^ such as tradition, gratitude or an expression of love, they felt responsible to take care for their loved one as long as possible at home in order to avoid admission to a nursing home. When the behavioural symptoms of dementia increased, family caregivers felt responsible to create a safe environment. Due to a lack of knowledge and/or experience, they did not always have enough competence to respond to these changing needs in a person-centred manner. This, in combination with previously observed safety risks and/or incidents, family caregivers tended to choose potentially harmful solutions such as forced hygiene, locking PLWD in their home or administering psychotropic medication. This is because family caregivers consider safety of great importance and were not always aware that the care they provided was involuntary and could have negative consequences.^23^ Dealing with these situations in combination with being 24/7 alert and concerned about what could happen was experienced as a stressful situation and hard to bear. Also, family caregivers did not always respond to these stressful situations by taking time for themselves or involving professional support. In some cases, they perceived professional help as a threat to their autonomy. Family caregivers were afraid to lose control of the caregiving process or that professionals would interfere in the caregiving process since they felt they knew their loved one the best.^31^ In addition, if professional help was involved, professionals did not sufficiently recognise and respond to their emotional care needs.^29^ All of these conflicts resulted in family caregivers’ finding the experience of dealing with safety versus autonomy in the care of PLWD as a complicated, stressful and exhaustive situation.^29^ These results underscore the importance that every member in the care triad recognise each other’s needs, seek insight into the interrelationship between family caregiver and PLWD,^30^ identify possible care conflicts and pursue dialogue with each other so that timely support and advice can be given to each other to prevent involuntary treatment.

Although all family caregivers experience care dilemmas as complicated, some deliberately choose not to intervene and accept possible (safety) risks. This can be explained not only by a difference in attitudes regarding involuntary treatment^22^ due to previous negative care experiences with the application of it, but also by the extent to which they feel supported by professional caregivers and their social network. As long as there are no safety issues or behavioural symptoms, family caregivers indicated that they respected the autonomy of the PLWD and wished to care for the PLWD themselves, without professional support. In the early stage of dementia, family caregivers do not acknowledge their needs and overestimate their capabilities because they struggle with accepting and adapting to their new role as caregiver.^32^ All of this highlights the importance of early interventions that inform, guide and support family caregivers on how to handle care dilemmas in a more person-centred manner.^31–33^

By increasing awareness through counselling such as by informing family caregivers about caregiver burden, behavioural problems, discussing alternatives and strengthening their social network (e.g. by attending support groups, involving formal and informal care support), family caregivers can be supported in the prevention and reduction of the underlying factors such as caregiver burden, lack of knowledge, skills and support that could lead to the application of involuntary treatment.^3,33–35^ District nurses play a pivotal role since they often are involved in the application of involuntary treatment.^21,36^ Therefore, they require education concerning alternative approaches as well as assistance in their own ethical reflection regarding the use of involuntary treatment.^37^ A multicomponent, person-centred, dementia care intervention is needed for district nurses so they can recognise the needs of PLWD and their caregivers and effectively support them. This multidisciplinary approach should focus on education, coaching and alternatives that can support both professional and family caregivers in discussing complex care dilemmas and making informed decisions regarding treatment.^38,39^ Further studies are needed to focus on the development and effectiveness of such approaches.

### Limitations and strengths

A limitation of our study was the relatively small sample size of 13 cases. In addition, 3 of the 10 participants provided information about earlier experiences and one could question the accuracy of their recollections due to the time gap between the actual experience and the interviews. Another limitation of this study was that involuntary treatment is a difficult concept to discuss. Family caregivers may not be aware of this term and, therefore, not recognise certain measures as involuntary. For this reason, we did not use the term ‘involuntary treatment’ in the interviews. Instead, we referred to care dilemmas in which the PLWD and family caregiver had different wishes, and care was provided against the PLWD’s will. Participants, however, were very willing to describe their experiences and provided detailed answers to our questions. This contributed to the richness and saturation of the data collected. A strength of this study was the use of purposive sampling, which led to a heterogeneous sample representative of family caregivers providing care for PLWD.

## Conclusion

The results from this study indicated that dealing with care dilemmas was experienced as complicated, stressful and exhausting. To prevent involuntary treatment, professional caregivers need to provide anticipatory guidance that supports family caregivers when caring for PLWD, especially when behavioural symptoms with safety implications emerge. In addition, family caregivers should be supported in finding the right balance between safety and autonomy and in handling their feelings of responsibility. Interventions are needed for both professional and family caregivers to acknowledge the ethically complex decision-making process in a more person-centred manner.
